# NbSOBIR1 Partitions Into Plasma Membrane Microdomains and Binds ER-Localized *Nb*RLP1

**DOI:** 10.3389/fpls.2021.721548

**Published:** 2021-09-01

**Authors:** Yi-Hua Li, Tai-Yu Ke, Wei-Che Shih, Ruey-Fen Liou, Chao-Wen Wang

**Affiliations:** ^1^Department of Plant Pathology and Microbiology, National Taiwan University, Taipei, Taiwan; ^2^Institute of Plant and Microbial Biology, Academia Sinica, Taipei, Taiwan

**Keywords:** ER, plasma membrane, microdomain, SOBIR1, pattern-triggered immunity, receptor-like protein, microbe-associated molecular pattern, *Phytophthora parasitica*

## Abstract

The receptor-like kinase Suppressor of BIR1 (SOBIR1) binds various receptor-like proteins (RLPs) that perceive microbe-associated molecular patterns (MAMPs) at the plasma membrane, which is thought to activate plant pattern-triggered immunity (PTI) against pathogen invasion. Despite its potentially crucial role, how SOBIR1 transmits immune signaling to ultimately elicit PTI remains largely unresolved. Herein, we report that a *Nicotiana benthamiana* gene *Nb*RLP1, like *Nb*SOBIR1, was highly induced upon *Phytophthora parasitica* infection. Intriguingly, *Nb*RLP1 is characterized as a receptor-like protein localizing to the endoplasmic reticulum (ER) membrane rather than the plasma membrane. Using bimolecular fluorescence complementation and affinity purification assays, we established that *Nb*RLP1 is likely to associate with *Nb*SOBIR1 through the contact between the ER and plasma membrane. We further found that *Nb*SOBIR1 at the plasma membrane partitions into mobile microdomains that undergo frequent lateral movement and internalization. Remarkably, the dynamics of *Nb*SOBIR1 microdomain is coupled to the remodeling of the cortical ER network. When *Nb*SOBIR1 microdomains were induced by the *P. parasitica* MAMP ParA1, tobacco cells overexpressing *Nb*RLP1 accelerated *Nb*SOBIR1 internalization. Overexpressing *Nb*RLP1 in tobacco further exaggerated the ParA1-induced necrosis. Together, these findings have prompted us to propose that ER and the ER-localized *Nb*RLP1 may play a role in transmitting plant immune signals by regulating *Nb*SOBIR1 internalization.

## Introduction

As a sessile organism, plants have evolved a series of defense mechanisms to protect themselves against pathogens. Plant cells sense potential pathogens through the function of pattern recognition receptors (PRRs) that recognize a wide range of microbe-associated molecular patterns (MAMPs) to ultimately elicit pattern-triggered immunity (PTI) (Jones and Dangl, 2006; Dodds and Rathjen, 2010). The onset of PTI can rapidly activate a series of downstream plant defense responses, including callose deposition, generation of reactive oxygen species (ROS), activation of mitogen-activated protein kinase cascade, induction of defense genes, and in some cases cell death (Boller and Felix, 2009; Yu et al., 2017).

Plant PRRs are plasma membrane-localized transmembrane protein receptors belonging to the family of receptor-like kinases (RLKs) or receptor-like proteins (RLPs), which differ in the presence or absence of intracellular kinase domains (Zipfel, 2014). The emerging evidence suggests that plant immune response is triggered upon the perception of MAMPs, accompanied by the association of selected PRRs with additional factors, such as BRI1-associated receptor kinase 1 (BAK1), also known as somatic embryogenesis receptor-like kinase 3 (SERK3), and other members of the SERK family to form multimeric kinase complex on the cell surface (Roux et al., 2011; Albert et al., 2020). Moreover, PRRs of the RLP type are known to constitutively form complexes with Suppressor of BIR1 (SOBIR1), an RLK containing a short leucine-rich repeat (LRR) ectodomain (Liebrand et al., 2013).

SOBIR1 was originally identified in *Arabidopsis thaliana* as a counterplayer of BAK1-interacting receptor-like kinase1 (BIR1) (Gao et al., 2009). When overexpressed, SOBIR1 constitutively activates cell death and plant immune responses. Recent studies further demonstrated that SOBIR1 is indispensable for PTI elicited by various MAMPs, including sclerotinia culture filtrate elicitor 1 (SCFE1) from *Sclerotinia sclerotiorum*, *Botrytis cinerea* endopolygalacturonase 1, *Phytophthora* elicitins, and *Phytophthora sojae* XEG1, all of which are perceived by PRRs of the LRR-RLP type (Zhang et al., 2013, 2014; Du et al., 2015; Wang et al., 2018). Thus, SOBIR1 appears to function as a common adaptor protein for various LRR-RLP PRRs and thus is thought to play a central role in plant immunity.

Recent studies based on high-resolution microscopy indicate that the immune and growth receptors form distinct nanodomains on the plasma membrane and these organized units potentially provide specificity for the downstream signaling events in plants (Bücherl et al., 2017). Nonetheless, how exactly the upstream perception event communicates with intracellular components during plant immune signaling remains largely unknown. Notably, a variety of organelles are found in close proximity with the plasma membrane in plant cells, such as the endoplasmic reticulum (ER). This organelle occupies a large volume in the cell and exerts multiple functions, including protein and lipid synthesis, calcium storage, vesicular trafficking, as well as biogenesis of other organelles (Phillips and Voeltz, 2016). Moreover, it is known to further compartmentalize into sheets and tubules, along with a variety of microdomains, to enable its versatile functions (English et al., 2009). In *Arabidopsis*, the ER makes contact with the plasma membrane through the ER–PM contact site (EPCS), with synaptotagmin 1 (SYT1) and vesicle-associated protein 27 (VAP27) as tethers that mediate EPCS formation (Perez-Sancho et al., 2016). The presence of membrane contact site (MCS) defines a unique feature of eukaryotes. More and more pieces of evidence have shown that MCS form transiently between two membrane compartments, creating a bridge for interorganelle communication, such as exchanges of metabolite, cellular stress response, membrane dynamics, and signaling (Prinz et al., 2020). However, to date, not much is known about the association of cellular organelles with the PRRs and their role in plant immune responses.

Previously, we demonstrated that tomato (*Solanum lycopersicum*) *Sl*SOBIR1 and *Sl*SOBIR1-like are required for the perception of the elicitin ParA1, a MAMP from the oomycete pathogen *Phytophthora parasitica*, and for plant defense against this pathogen (Peng et al., 2015). We documented that *Sl*SOBIR1 is translocated from the plasma membrane to endosomes in response to ParA1 treatment, which suggests that *Sl*SOBIR1 endocytosis is coupled to the plant immune signaling. To further tackle the function and regulation of SOBIR1, we took the biochemical approach to look for SOBIR1-interacting proteins. In this study, we report a novel RLP from *Nicotiana benthamiana*, named *Nb*RLP1 that encodes an unconventional RLP protein residing within the ER membrane, but not the plasma membrane. By using bimolecular fluorescence complementation and tandem-affinity purification, we validated the interaction between *Nb*RLP1 and *Nb*SOBIR1. In addition, we observed that *Nb*SOBIR1 formed microdomains on the plasma membrane whose dynamics is coupled to the remodeling of the ER. Further evidence supports that *Nb*RLP1 downregulates the number of *Nb*SOBIR1 microdomains and promotes *Nb*SOBIR1 internalization in the presence of ParA1 that triggers partitioning of *Nb*SOBIR1 into the microdomains. As overexpressing *Nb*RLP1 exaggerated the ParA1-induced necrosis in plants, we propose that ER and unconventional RLPs in the ER, such as *Nb*RLP1, are engaged in SOBIR1-mediated plant immunity through mediating MAMP-induced SOBIR1 internalization.

## Results

### Like *NbSOBIR1*, *NbRLP1* Is Highly Induced Upon *Phytophthora parasitica* Infection

**FIGURE 1:**
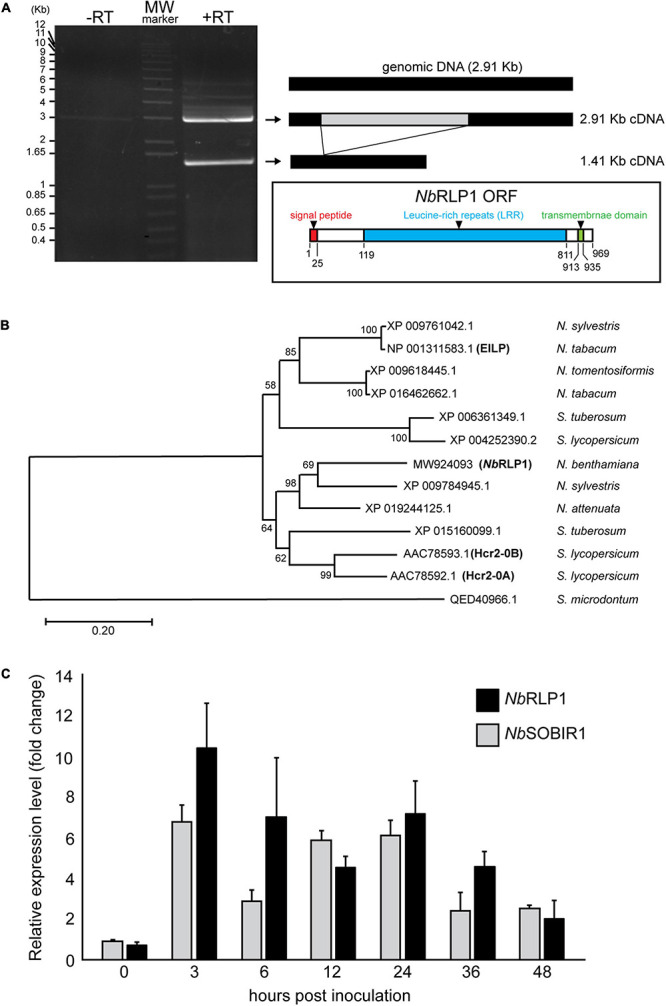
The expression of *Nb*RLP1 is induced in response to infection by *Phytophthora parasitica*. **(A)** *Nb*RLP1 is alternatively spliced. Reverse transcriptase-polymerase chain reaction (RT-PCR) gave rise to two amplified products with respective length of 1.41 and 2.91 Kb (+ RT), which were not detected in the absence of reverse transcriptase (-RT). The 2.91-Kb gene, named *NbRLP1* (MW924093) encodes a 969-amino acid protein containing a signal peptide (predicted by SignalP), an LRR domain (predicted by InterPro), and a transmembrane domain (predicted by TMHMM) as illustrated in the inset. **(B)** Phylogenetic analysis of *Nb*RLP1. Multiple sequence alignment of *Nb*RLP1 and its homologs from *Nicotiana* and *Solanum* retrieved from the protein database of the National Center for Biological information (NCBI) was performed by using ClustalX. The phylogenetic tree was constructed by employing the maximum likelihood method, followed by bootstrap analysis with 1,000 pseudo-replicates. **(C)** The expression of the *Nb*RLP1 and *Nb*SOBIR1 is upregulated in response to *P. parasitica* infection. At the indicated hours post *P. parasitica* zoospore inoculation of *Nicotiana benthamiana*, total RNAs were isolated and analyzed by quantitative reverse transcription PCR (RT-PCR). Data are presented as fold-change relative to mock treatment of the same time point. Values are means (±SE) from three independent experiments.

*Sl*SOBIR1 is involved in PTI response to the *P. parasitica* MAMP termed ParA1 (Peng et al., 2015). To identify proteins associated with *Sl*SOBIR1 during the process, we treated *Nicotiana benthamiana* harboring *Sl*SOBIR1-GFP with ParA1, followed by immunoprecipitation with anti-GFP antibody. Among a collection of putative *Sl*SOBIR1-interacting proteins, we selected one of the major candidates, NbS0003586g0006.1 [Niben.0.4.4., Sol genetic network (SGN^1^)] for further analysis. In contrast to no signal in the control without reverse transcriptase, two major transcripts were detected by reverse transcriptase-PCR reaction (Figure 1A). One is a 2.91 Kb cDNA (GenBank Accession number MW924093), which corresponds to the exact genomic DNA sequence of *NbRLP1*, and the other shorter cDNA of 1.41 Kb lacks the putative intron sequence (MW924094), reflecting a spliced form of *NbRLP1* (Supplementary Table 1). We focused on the 2.91-Kb gene, named NbRLP1 hereafter, that encodes a 969-amino acid protein with a characteristic of RLPs (Figure 1A).

Phylogenetic analysis of *NbRLP1* and its homologs from Solanaceae retrieved from the NCBI databases indicates that XP_009784945 of *Nicotiana sylvestris* and XP_019244125 of *Nicotiana attenuata* are close homologs of *NbRLP1* (Figure 1B). These genes along with AAC78592 (Hcr2-0A) and AAC78593 (Hcr2-0B), two homologs of tomato disease resistance gene, Cf-5 (Dixon et al., 1998), form a clade distinct from that encompassing elicitor-inducible EILP of *Nicotiana tabacum* (Takemoto et al., 2000). As well, these two clades are distinguished from ELR of *Solanum microdontum* (QED40966), an RLP involved in the recognition of *Phytophthora* elicitins (Du et al., 2015).

To know whether *NbRLP1* is induced by pathogen infection, we infected *N. benthamiana* with *P. parasitica* zoospores and performed quantitative RT-PCR, with the expression level of elongation factor 1 alpha (*NbEF1a*) serving as an internal control. The results showed that the expression of *NbRLP1*, like *NbSOBIR1*, was largely induced 3 h post-inoculation (hpi) and persists high expression through later infection stage to at least 36 hpi (Figure 1C). Collectively, these data imply a potential role of *Nb*RLP1 for plant defense responses.

### *Nb*RLP1 Is a Transmembrane Protein Localized to the ER

**FIGURE 2:**
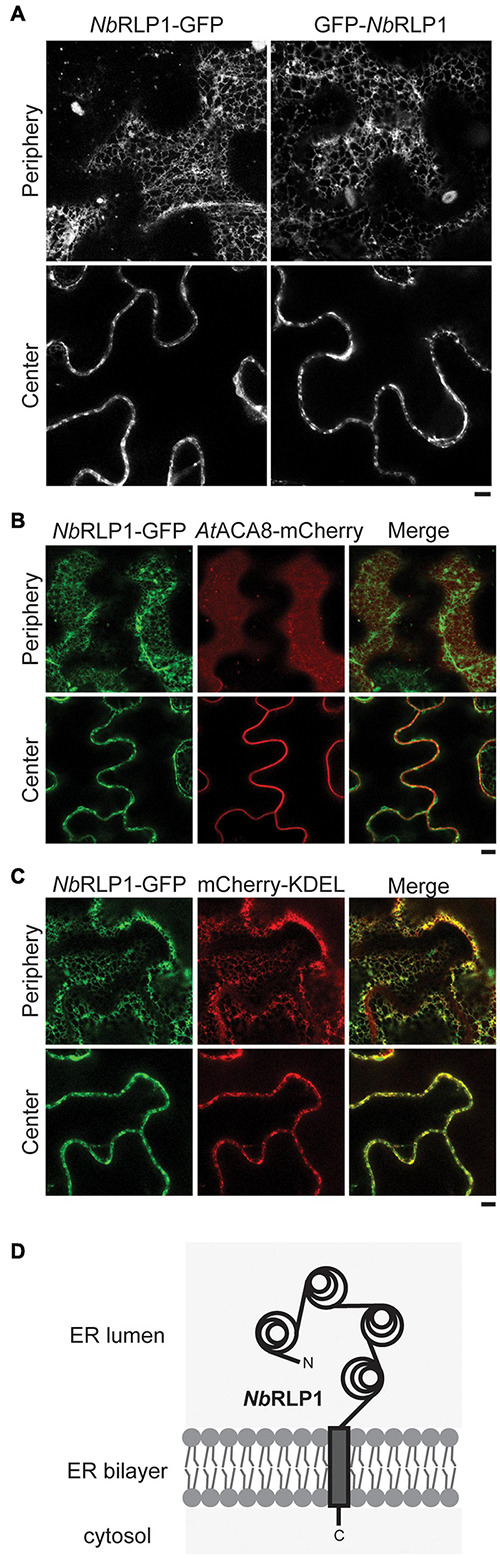
*Nb*RLP1-GFP resides in the endoplasmic reticulum (ER) membrane. **(A)** Still images of *Nb*RLP1-GFP or GFP-*Nb*RLP1 taken from the periphery or the center of the same *Nicotiana benthamiana* leaves by Zeiss LSM880 confocal microscope. Scale bar, 5 μm. **(B)** Same as A, except that *Nb*RLP1-GFP and *At*ACA8-mCherry are coexpressed in plants and imaged. Also shown are the corresponding merged images. **(C)** Same as B, except that *Nb*RLP1-GFP and mCherry-KDEL are coexpressed in plants and imaged. **(D)** A scheme depicting *Nb*RLP1 topology in the ER based on TMHMM.

To protect against pathogens, plant cells possess a variety of transmembrane RLKs and RLPs on the plasma membranes, many of which contain extracellular LRR domains to enable ligand recognition. Intriguingly, when we analyzed *Nb*RLP1 localization by the use of GFP fused to either amino- or carboxyl-terminus of *Nb*RLP1 for fluorescence microscopy, we observed a distribution pattern of network and/or multiple puncta near the cell cortex (Figure 2A). As it resembles the plant ER network, we next examined cells coexpressing *Nb*RLP1-GFP with the plasma membrane marker *At*ACA8-mCherry (Figure 2B) or the ER marker mCherry-KDEL (Figure 2C). The results showed that *Nb*RLP1-GFP localization is in large agreement with the luminal ER protein mCherry-KDEL but not with the evenly distributed *At*ACA8-mCherry signal of the plasma membrane. Thus, we conclude that *Nb*RLP1 is an unconventional RLP localizing to the ER rather than the plasma membrane. Based on sequence prediction results, *Nb*RLP1 is likely to adapt the standard type I transmembrane protein topology in the ER as depicted in Figure 2D. The main moiety of this protein, containing LRR, is in the ER lumen, and the protein also contains a single spanning transmembrane helix and a short cytoplasmic tail.

### *Nb*RLP1 Interacts With *Nb*SOBIR1

**FIGURE 3:**
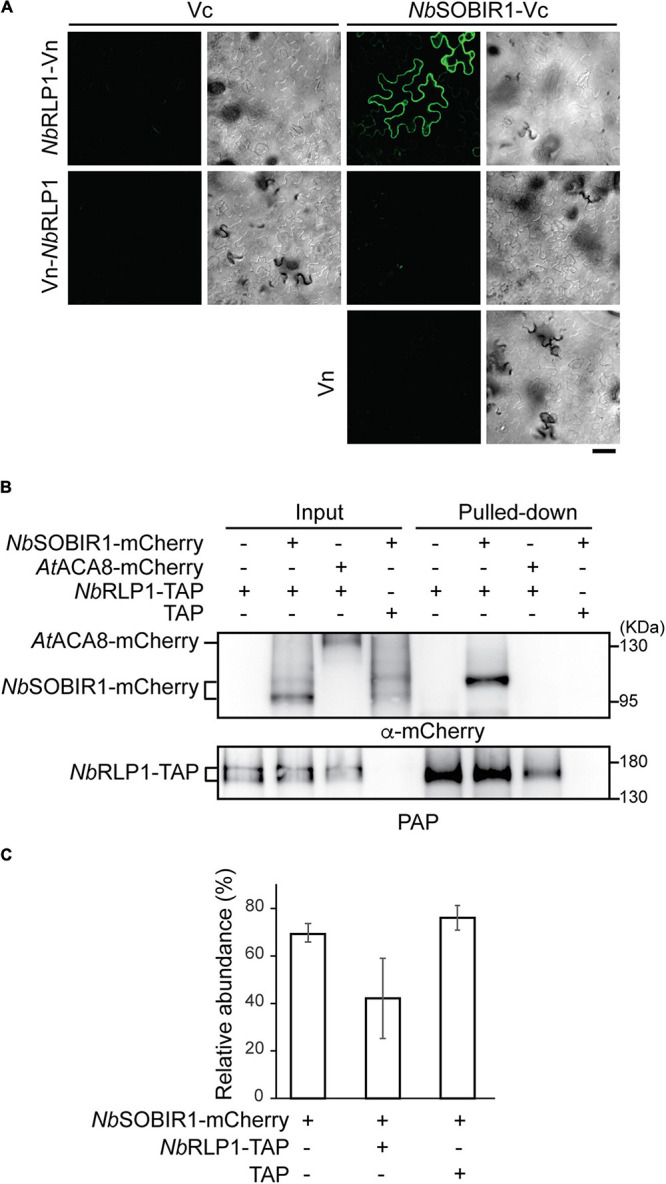
*Nb*RLP1 interacts with *Nb*SOBIR1. **(A)** The carboxyl half of Venus protein (Vc) alone or fused to *Nb*SOBIR1 was coexpressed with the amino half of Venus (Vn) alone or fused to *Nb*RLP1 as indicated on the *Nicotiana benthamiana* leaves. Images were taken by Zeiss LSM880 confocal microscope. Complemented Venus fluorescence and differential interference contrast (DIC) images of the same area are shown. Scale bar, 40 μm. **(B)** *N. benthamiana* leaves expressing *Nb*SOBIR1-mCherry, *At*ACA8-mCherry, *Nb*RLP1-TAP, or TAP as indicated were harvested and lyzed. The lysate (input) was subjected to pull-down by IgG Sepharose as described in the Materials and Methods section. The input and the bound (pulled-down) fractions were subjected to SDS-PAGE followed by the Western blot analysis with the use of anti-mCherry and PAP antibodies. **(C)** *N. benthamiana* leaves expressing *Nb*SOBIR1-mCherry, *Nb*RLP1-TAP, or TAP as indicated were lyzed and *Nb*SOBIR1-mCherry proteins in the lysate were subjected to SDS-PAGE followed by Western blot analysis with anti-mCherry antibody. The ratio of the upper *Nb*SOBIR1 band (with higher molecular mass) relative to the total *Nb*SOBIR1 (combining both the upper and lower bands of *Nb*SOBIR1) was plotted and compared. Data from three experimental repeats are shown as means (± SD).

To consolidate the potential relationship between *Nb*RLP1 and *Nb*SOBIR1, we first employed the bimolecular fluorescence complementation (BiFC) assay, known to provide *in situ* interacting information *in planta*. Our results indicate that *Nb*RLP1-Vn, but not Vn-*Nb*RLP1, interacts with *Nb*SOBIR1-Vc in plants, thus showing Venus YFP fluorescence, in contrast to no or dim signals of various negative controls (Figure 3A). The fact that only Vn fused to *Nb*RLP1 at the C-terminus but not to N-terminus gave a positive BiFC signal, which is consistent with the topological prediction of *Nb*RLP1 in the ER (Figure 2D). Since *Nb*RLP1 is an ER protein and *Nb*SOBIR1 is a plasma membrane protein, we hypothesize that their interaction most likely is achieved through close proximity between the ER and plasma membrane. To know whether *Nb*RLP1 may partition into the previously characterized EPCS, *Nb*RLP1-GFP was coexpressed with the EPCS tether *At*SYT1-mCherry. Indeed, we observed that signals of the two proteins colocalized at the cell periphery (Supplementary Figure 1). Thus, these results imply that the ER-localized *Nb*RLP1 might interact with the plasma membrane-localized *Nb*SOBIR1 protein *in vivo*, likely at a position where cortical ER and the plasma membrane join in physical proximity, such as the EPCS tethered by *At*SYT1-mCherry.

We next performed biochemical studies to further strengthen the notion that *Nb*RLP1 interacts with *Nb*SOBIR1. To achieve this, we generated an *Nb*RLP1 harboring a TAP tag (an S-tag followed by a protein A tag) at its C-terminus and asked whether the fusion protein (termed *Nb*RLP1-TAP) binds *Nb*SOBIR1-mCherry in detergent-solubilized plant extracts. To unambiguously judge the interaction, we also tested whether *Nb*RLP1-TAP binds another plasma membrane protein *At*ACA8-mCherry. Our results showed that *Nb*SOBIR1-mCherry, but not *At*ACA8-mCherry, was pulled down by *Nb*RLP1-TAP, examined by Western blotting using an antibody against mCherry (Figure 3B) as well as mass spectrometry (not shown). In contrast, *Nb*SOBIR1 signal has never been detected in the pulled-down fractions derived from TAP-alone expressing samples, indicative of specificity. Intriguingly, Western blotting data revealed two major forms of *Nb*SOBIR1-mCherry with a size difference of ∼8.5 kD in the inputs; however, only *Nb*SOBIR1-mCherry with the higher molecular weight was pulled down by *Nb*RLP1-TAP (Figure 3B). Notably, the expression of *Nb*RLP1-TAP but not TAP alone in plants reduced the proportion of the larger molecular weight form of *Nb*SOBIR1 in the steady state (Figure 3C), implying that *Nb*RLP1-TAP not only binds to this unique form of *Nb*SOBIR1 but may further reduce its expression or accelerate its turnover.

The BiFC data supports that the short tail of *Nb*RLP1 fused with Vn interacts with the cytoplasmic domain of *Nb*SOBIR1 fused with Vc. To know the contribution of the N-terminal LRR domain and the short C-terminal cytoplasmic tail of *Nb*RLP1 to its interaction with *Nb*SOBIR1, we also prepared different truncated versions of *Nb*RLP1. Analysis by using the biochemical pulled-down assay indicates *Nb*RLP1-ΔC-TAP with the short cytoplasmic tail removed still bound with *Nb*SOBIR1 (Supplementary Figure 2A). In contrast, *Nb*RLP1-ΔN-TAP, having signal peptide but lacking the large LRR domain, is bound to *Nb*SOBIR1 with reduced affinity (Supplementary Figure 2A). Although the *Nb*RLP1 with cytoplasmic tail and a transmembrane anchor seems to be sufficient for binding with *Nb*SOBIR1, the result implies that other proteins in the ER or the plasma membrane may bridge or stabilize the interaction between *Nb*RLP1 and *Nb*SOBIR1 (Supplementary Figure 2B). Collectively, both *in vivo* and *in vitro* data support the notion that the ER-localized *Nb*RLP1 interacts with the plasma membrane-localized *Nb*SOBIR1.

### *Nb*SOBIR1 Is Partitioned Into a Dynamic Microdomain on the Plasma Membrane

**FIGURE 4:**
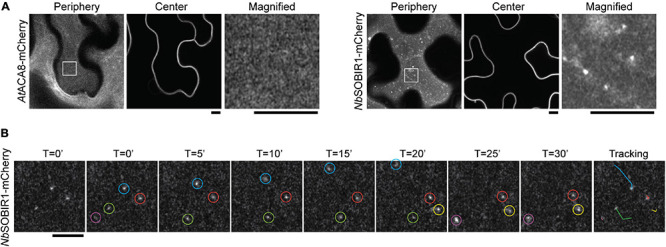
*Nb*SOBIR1-mCherry forms microdomains in the plant plasma membrane. **(A)***Nicotiana benthamiana* leaves expressing *At*ACA8-mCherry or *Nb*SOBIR1-mCherry were imaged at the periphery or center sections using Zeiss LSM880 confocal microscope with Airyscan. The box area in the image of the periphery section was enlarged and displayed to show the microdomains of *Nb*SOBIR1 that were not detectable on *At*ACA8-mCherry images. Scale bar, 5 μm. **(B)** *N. benthamiana* leaves expressing *Nb*SOBIR1-mCherry was subjected to time-lapse microscopy by Zeiss LSM880 confocal microscope with the Airyscan. The total image acquisition length was 16 min with a time interval of 5 s (Supplementary Movie 1). Representative area was selected for particle tracking analysis using the ImageJ, and the image of each time point is shown. Five microdomains, each circled by different colors were identified in the displayed image and their tracks are shown in the right-most image.

Having established that *Nb*RLP1 interacts with *Nb*SOBIR1, we next explored the potential regulatory mechanism underlying their interplay *in planta*. The observation of two major forms of *Nb*SOBIR1-mCherry, while only one form is capable of binding with and being regulated by *Nb*RLP1, prompted us to investigate whether *Nb*SOBIR1 may associate with distinct structures on the plasma membrane. We thus performed confocal microscopy with Airyscan to gain insights into *Nb*SOBIR1-mCherry localization on the plasma membrane. Interestingly, in addition to the dispersed signal along the plasma membrane, *Nb*SOBIR1-mCherry was identified in several punctate structures, which was not observed when another plasma membrane protein, *At*ACA8-mCherry, was subjected to imaging by the Airyscan microscopy (Figure 4A). Thereby, we suggest that a portion of *Nb*SOBIR1 is likely partitioned into microdomains on the plasma membrane.

To further understand the nature of *Nb*SOBIR1-mCherry microdomains on the plasma membrane, we imaged these fine structures using time-lapse microscopy with a time interval of 5 s. The results clearly showed that these plasma membrane microdomains of *Nb*SOBIR1 are mobile structures, although a small portion of them appeared relatively more static (Figure 4B and Supplementary Movie 1). Particle tracking analysis data indicated that the *Nb*SOBIR1-mCherry microdomains can move laterally, showing an irregular pattern (Figure 4B). During the course of our analysis, we also observed the disappearance (Figure 4B, green circle) and emergence (Figure 4B, yellow circle) of these microdomains on the plasma membrane. In either case, it appears to involve a quick biogenesis and turnover process, detected within the time interval of 5 s. Considering SOBIR1 functions as an adaptor protein that assembled together with various LRR-RLPs into a receptor complex on the plasma membrane awaiting ligand binding, partitioning of SOBIR1 into microdomains may serve as a platform to facilitate such a process. Overall, these observations have led us to hypothesize that the dynamics of these microdomains of *Nb*SOBIR1 is a regulated process and that the microdomain structure may represent one of the functional forms of *Nb*SOBIR1.

### ER Remodeling Is Coupled to the Dynamics of *Nb*SOBIR1 Microdomains

**FIGURE 5:**
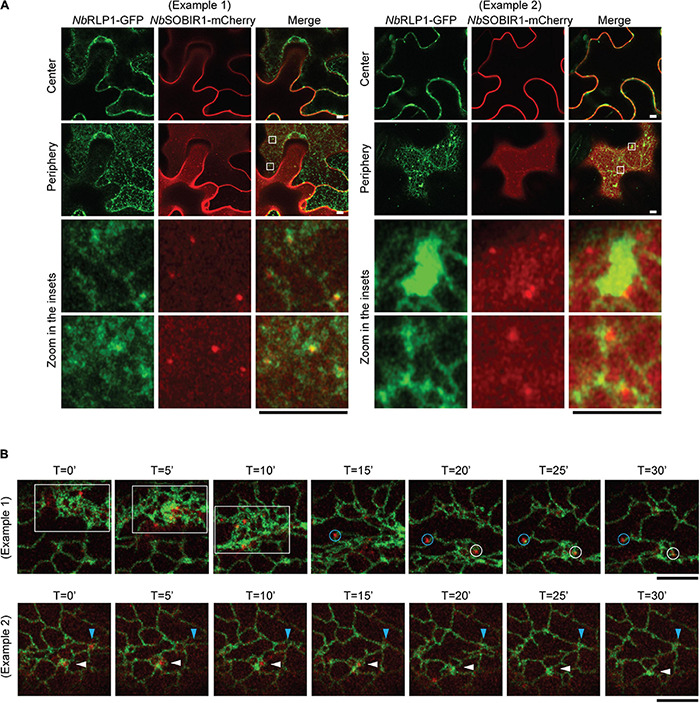
The *Nb*SOBIR1 microdomains in the plasma membrane move as the cortical ER changes the morphology**. (A)** *Nicotiana benthamiana* leaves expressing *Nb*SOBIR1-mCherry and *Nb*RLP1-GFP were subjected to confocal microscopy by Zeiss LSM880 with use of Airyscan. The periphery and center sections of the epidermal cell of the same leaf were imaged, and the insets were enlarged to reveal the association between *Nb*SOBIR1-mCherry and *Nb*RLP1-GFP. Examples 1 and 2 show different degrees of ER tubules and sheets marked by *Nb*RLP1-GFP to reveal how *Nb*SOBIR1 microdomains are associated with the ER. **(B)** *N. benthamiana* leaves expressing *Nb*SOBIR1-mCherry and *Nb*RLP1-GFP were subjected to time-lapse microscopy by Zeiss LSM880 confocal microscope with use of Airyscan. The total image acquisition length was 16 min with a time interval of 5 s (Supplementary Movie 2). Representative area was selected for display by time course. (Example 1) White box area, containing several *Nb*SOBIR1 microdomains, represents movement of these structures after a quick remodeling of the ER harboring high degree of sheets. Blue and white circles denote two *Nb*SOBIR1 microdomains associated with ER tubules. (Example 2) Blue and white arrowheads denote two *Nb*SOBIR1 microdomains that are internalized from the cell surface as the ER transformed from tubules to sheets. Scale bar, 5 μm.

Given that the aforementioned *Nb*SOBIR1-*Nb*RLP1 interaction depends on close proximity between the ER and the plasma membrane, we further asked whether these *Nb*SOBIR1 microdomains may have an association with the ER. The eukaryotic ER is organized into a complicated network containing various degrees of sheets and tubules (Shibata et al., 2006; English et al., 2009). The highly dynamic ER disperses throughout the entire cytoplasm and is thought to fine-tune cell physiology to cope with environmental cues. When the localization of *Nb*RLP1-GFP was compared with *Nb*SOBIR1-mCherry, we noticed that *Nb*SOBIR1-mCherry was either associated with the edge of the ER sheets or surrounded by the three-way junction of the ER tubules labeled with *Nb*RLP1 at the cell periphery (Figure 5A).

Employing time-lapse microscopy, we observed that ER labeled by *Nb*RLP1-GFP constantly undergoes morphological remodeling (Figure 5B and Supplementary Movie 2). Most importantly, the lateral movement of *Nb*SOBIR1 microdomains was correlated with the dynamic changes of the ER, as the ER labeled with *Nb*RLP1 always associated with the movement of the *Nb*SOBIR1 microdomains (Figure 5B, blue and white circles, and Supplementary Movie 2). The correlation of the *Nb*SOBIR1 microdomains with the cortical ER suggests that these microdomain structures are likely where *Nb*SOBIR1 makes contact with *Nb*RLP1 at the first place in their native state. Intriguingly, we observed that *Nb*SOBIR1-mCherry microdomains diminished in the condition when a wave of ER sheets transit through the cell cortex during ER remodeling (Figure 5B, white box). In addition, we observed *Nb*SOBIR1 microdomains originally surrounded by ER tubules disappeared in the next time point with the same area being replaced with ER sheets (Figure 5B, white and blue arrowheads). Collectively, the evidence in which *Nb*SOBIR1 microdomain dynamics is correlated with ER remodeling raises an interesting possibility that ER may contribute to *Nb*SOBIR1-mediated endocytosis and/or plant immunity.

### *Nb*RLP1 Overexpression Promotes *Nb*SOBIR1 Endocytosis Upon ParA1 Elicitin Treatment

**FIGURE 6:**
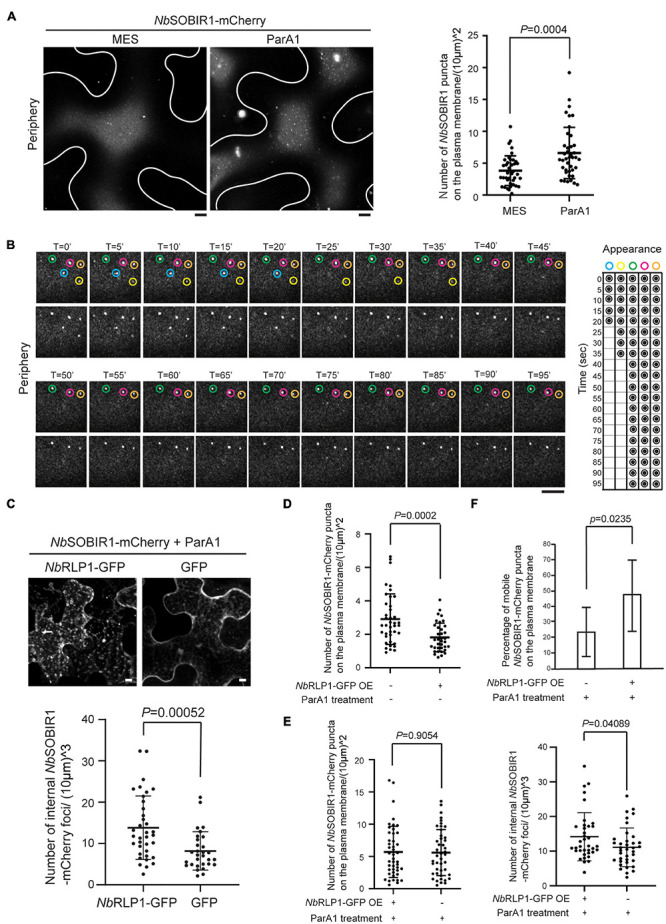
*Nb*RLP1-GFP overexpression promotes *Nb*SOBIR1-mCherry endocytosis upon ParA1 elicitin treatment. **(A)***Nicotiana benthamiana* leaves expressing *Nb*SOBIR1-mCherry were infiltrated with MES buffer alone (control) or 0.3 μMParA1 in MES buffer. After 30 min, the leaves were subjected to microscopy, focusing only on the periphery of the leaf epidermal cell, using the Zeiss LSM880 confocal microscope with an Airyscan. Representative images are shown on the left panel with an outline of the cell, and the quantification data using the scatter plot is shown on the right. Statistics is carried out by the two-tailed student’s *t*-test and the *p*-value is displayed. **(B)** *N. benthamiana* leaves expressing *Nb*SOBIR1-mCherry and *Nb*RLP1-GFP were infiltrated with 0.3 μM ParA1 in MES buffer. After 30 min, leaves were subjected to time-lapse microscopy using Zeiss LSM880 confocal microscope with Airyscan. The total image acquisition length was 16 min with a time interval of 5 s (Supplementary Movie 4). Representative area was selected for display by time course, and the appearance and absence of each microdomain at each displayed time point was summarized on the right. Blue and yellow circles mark two *Nb*SOBIR1 microdomains with a larger size undergoing endocytosis during the imaging time frame. Green, pink, and orange circles mark *Nb*SOBIR1 microdomains with a larger size that remain static during the imaging time frame. **(C)** *N. benthamiana* leaves expressing *Nb*SOBIR1-mCherry and *Nb*RLP1-GFP or GFP alone were infiltrated with 0.3 μM ParA1 in MES buffer. After 30 min, leaves were subjected to microscopy with five Z stack for a total of 8.20 mm stack size using Zeiss LSM 510 confocal microscope. The maximal projection images are shown. The number of internal *Nb*SOBIR1-mCherry puncta was quantified as described in the Materials and Methods section and displayed using a scatter plot. Statistical analysis was carried out with the two-tailed student’s *t*-test and the *p*-value is shown. **(D)** *N. benthamiana* leaves expressing *Nb*SOBIR1-mCherry alone or coexpressed with *Nb*RLP1-GFP were subjected to microscopy using the Zeiss LSM880 confocal microscope with an Airyscan. Microdomain number was quantified as described in the Materials and Methods section and shown as scatter plots. Statistics is carried out with the two-tailed student’s *t*-test and the *p*-value is displayed. **(E)** *N. benthamiana* leaves expressing NbSOBIR1-mCherry alone or coexpressing with NbRLP1-GFP were treated with 0.3 μM ParA1. After 30 min, leaves were subjected to microscopy by Zeiss LSM880 confocal microscope with the use of Airyscan. Microdomain number (left) was quantified as in **(D)**. The same leaves were subjected to Zeiss LSM880 confocal imaging and quantified for internal NbSOBIR1-mCherry foci number (right) as in **(C)**. Statistics is carried out with the two-tailed student’s *t*-test and the *p*-values are displayed. **(F)** *N. benthamiana* leaves expressing NbSOBIR1-mCherry alone or coexpressing with NbRLP1-GFP were infiltrated with 0.3 μM ParA1. After 30 min, leaves were subjected to time-lapse microscopy by Zeiss LSM880 confocal microscope with the use of Airyscan. The ParA1-induced microdomains were quantified if their size was bigger than 0.4 mm in diameter. A total of 10 cells for each treatment was quantified within an imaging timeframe of 60 s and data are shown as percentage (number of mobile puncta/number of total puncta). Statistics is carried out with the two-tailed student’s *t*-test and the *p*-value is displayed.

In our previous paper, we have established that *Sl*SOBIR1 endocytosis is triggered by the perception of an oomycete MAMP termed, ParA1. If the *Nb*SOBIR1 microdomain defines a functional unit for the protein, we suspect that this structure is likely responsive to ParA1. Accordingly, we treated the *Nb*SOBIR1-overexpressing plants with ParA1 to compare with the treatment with MES buffer, followed by Airyscan confocal microscopy to monitor the extent of *Nb*SOBIR1 microdomain formation on the plasma membrane. Our results clearly showed that the number of *Nb*SOBIR1 microdomain formed on the plasma membrane increased significantly in the ParA1-treated condition compared to the MES control, and the quantification results based on a number of cells examined further support the notion (Figure 6A). Remarkably, ParA1 treatment resulted in the formation of more *Nb*SOBIR1-mCherry microdomains of a larger size which appeared more static (Figure 6A and Supplementary Movie 3). Time-lapse microscopy experiments performed with *N. benthamiana* plants coexpressing *Nb*RLP1 identified that these larger *Nb*SOBIR1-mCherry puncta though relatively more static can become diminished and/or disappear quickly (Figure 6B, blue and yellow circles, and Supplementary Movie 4), representing the occurrence of either endocytosis or diffusion upon ParA1 perception. While focusing on the focal plane containing the ParA1-induced static *Nb*SOBIR1 microdomains, time-lapse microscopy revealed that these structures move inward from the cell cortex, which supports that endocytosis of *Nb*SOBIR1 occurs directly through the microdomain structures upon ParA1 perception (Supplementary Figure 3).

We further investigated the potential role of *Nb*RLP1 for *Nb*SOBIR1 endocytosis under ParA1 treatment based on a condition we reported previously (Peng et al., 2015). First, we asked whether coexpressing *Nb*RLP1 and *Nb*SOBIR1 affect *Nb*SOBIR1 endocytosis in response to ParA1 treatment. Compared to the control coexpressing GFP and *Nb*SOBIR1-mCherry, more intracellular vesicles harboring *Nb*SOBIR1-mCherry were observed on tobacco leaves coexpressing *Nb*RLP1-GFP and *Nb*SOBIR1-mCherry in response to ParA1 treatment (Figure 6C). Given that ParA1 treatment induced the formation of *Nb*SOBIR1 microdomains and that *Nb*RLP1 overexpression promoted ParA1-induced *Nb*SOBIR1 endocytosis, it seems plausible to predict that *Nb*RLP1 overexpression may facilitate *Nb*SOBIR1 endocytosis, thereby reducing the number of *Nb*SOBIR1 microdomains on the plasma membrane. As shown in Figure 6D, in the absence of ParA1, a condition when fewer *Nb*SOBIR1 microdomains were visualized, *Nb*RLP1 overexpression caused a significant reduction in the number of *Nb*SOBIR1 microdomains present on the plasma membrane. In the presence of ParA1, although overexpressing *Nb*RLP1 did not significantly reduce the number of *Nb*SOBIR1 microdomains on the plasma membrane (Figure 6E, left panel), it appears to accelerate the mobility of *Nb*SOBIR1 microdomains as reflected by the higher percentage of mobile *Nb*SOBIR1 puncta detected on the plasma membrane (Figure 6F). Moreover, overexpressing *Nb*RLP1 led to the detection of more *Nb*SOBIR1-mCherry-labeled structures inside the plant cells, indicative of more active *Nb*SOBIR1 endocytosis (Figure 6E, right panel). Collectively, these data have led us to propose that the ParA1 elicitin-inducible *Nb*SOBIR1 microdomain formed on the plasma membrane likely represents an active unit of *Nb*SOBIR1 for subsequent receptor-mediated endocytosis and that *Nb*RLP1 might act as a positive regulator for *Nb*SOBIR1 internalization.

### Overexpressing *Nb*RLP1 Exaggerated the ParA1-Induced Necrosis in Plants

**FIGURE 7:**
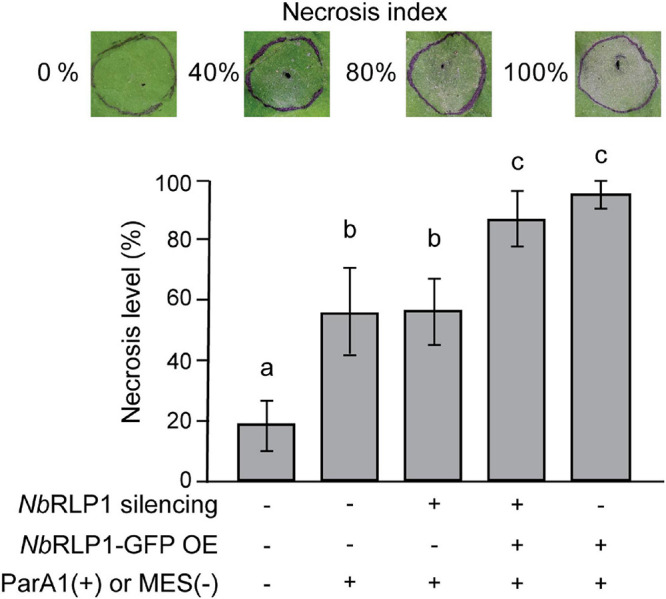
*Nb*RLP1 overexpression exaggerated the ParA1-induced necrosis on *Nicotiana benthamiana* leaves. *N. benthamiana* leaves that are either silenced for *Nb*RLP1 expression or overexpressing *Nb*RLP1 are infiltrated with 0.3 μM ParA1 or 10 mM MES (control) as indicated. After 24 h, the development of cell death was analyzed based on the representative necrosis index, defined by the percentage of necrosis area over the infiltrated area. The experiment has been repeated four times with similar results, and only one set of the experimental result is shown. Values are means (± SD) of 18 biological repeats. Different letters indicate significant differences based on LSD (*p* < 0.01).

Our data have supported that the unconventional *Nb*RLP1 in ER underlies part of the regulatory network for *Nb*SOBIR1, the essential adaptor for various LRR-RLPs, and thus it seems likely that *Nb*RLP1 might contribute to the *Nb*SOBIR1-associated PTI. To explore this possibility, we analyzed the effect of *Nb*RLP1 overexpression on ParA1-induced necrosis, a downstream output of plant immune response. Consistent with our previous findings, ParA1 induced the formation of necrotic lesions on *N. benthamiana* leaves at 24 h post-treatment (Figure 7). *Nb*RLP1 silencing under either *Nb*RLP1 endogenous or overexpressing conditions did not cause a significant change for the ParA1-induced necrosis on *N. benthamiana* leaves when compared to the control group. However, when ParA1 treatment was carried out in the *N. benthamiana* plants overexpressing *Nb*RLP1-GFP, a condition when more *Nb*SOBIR1 endocytic vesicles were observed (Figure 6C), we observed that necrotic lesions on *N. benthamiana* leaves were more exaggerated as evaluated by ANOVA. Altogether, these results support that *Nb*RLP1 acting as a regulator lies within the repertoire of *Nb*SOBIR1-mediated PTI.

## Discussion

To combat pathogen infection, plants have evolved PRRs, which detect MAMPs at the cell surface to elicit PTI. Studies in various plant systems demonstrated that SOBIR1 plays a central role in PTI involving various PRRs of the LRR-RLP type (Tang et al., 2017; Domazakis et al., 2018). SOBIR1 moves from the plasma membrane to endocytic vesicles *via* endocytosis in response to MAMP treatment (Liebrand et al., 2013; Peng et al., 2015). However, how exactly SOBIR1 functions in plant immune response remains largely elusive.

Located at the outermost boundary of the cell as to communicate with the environment, the plasma membrane is known to be compartmentalized into subdomains, where immune complexes may be formed even in the absence of matching ligand, enabling rapid transmission and activation of diverse immune responses (Jarsch et al., 2014; Somssich et al., 2015; Bücherl et al., 2017). In this study, we report the identification of dynamic microdomains of *Nb*SOBIR1 on the plasma membrane, which reinforces the important concept of nano- and micro-domains in plant immune response.

We document in this study that the ParA1 elicitin treatment induced both in the number and size of *Nb*SOBIR1-labeled microdomaind, formed on the plasma membrane (Figure 6A), concomitant with the induction of *Nb*SOBIR1 internalization, reflecting that the protein forms clusters in response to elicitin perception in *N. benthamiana*. The importance of nano- and micro-domains for plant innate immunity has been demonstrated in a number of recent studies. In the response of rice (*Oryza sativa*) to chitin elicitor, microdomains are required for the dynamics of the plasma membrane-anchored Rac/ROP small GTPase Rac1 (a molecular switch in defense signaling) and NADPH oxidase-encoding respiratory burst oxidase homologs (RBOHs) (Nagano et al., 2016). As well, the immune receptor FLS2 required for the perception of flg22 also localizes to the plasma membrane nanodomains of *Arabidopsis* (Bücherl et al., 2017). Consistently, *Nb*SOBIR1 microdomain we discovered here plays a crucial role in the PTI regulated through PRRs of the RLP type.

Our findings uncovered *Nb*RLP1 as a novel player in underlying the network of *Nb*SOBIR1-mediated PTI response, which led to a yet unexplored area regarding the engagement of ER during *Nb*SOBIR1 endocytosis and/or plant immunity. Using the *in vivo* BiFC assay and the *in vitro* pulled-down assay, we document in this study that the plasma membrane-localized *Nb*SOBIR1 interacts with the ER-localized transmembrane protein *Nb*RLP1, whose LRR domain resides within the ER lumen (Figure 3). This result also predicts that *Nb*RLP1 and *Nb*SOBIR1 might interact *via* a close association between the ER and the plasma membrane, such as EPCS or the ER-endosomes interface (Dong et al., 2016). However, BiFC fluorescence may emit artificially due to close proximity of target proteins, and the assay results in the irreversible formation of fluorescent proteins, thus limiting its applications to further explore the dynamics and transient interactions between *Nb*RLP1 and *Nb*SOBIR1 (Shyu and Hu, 2008). In addition, as reported by Tao et al. (2019), the BiFC assay in plants has the tendency to artificially induce membrane contact especially when involving the overexpression of two proteins which might interact with ER and plasma membrane interfaces. Thus, where exactly does the interaction between *Nb*RLP1 and *Nb*SOBIR1 occur awaits further investigation.

To compensate for this limitation, we studied the localization and dynamics of *Nb*RLP1 and *Nb*SOBIR1 in plants by transient overexpression followed by confocal microscopy. Remarkably, our findings clearly uncovered that the dynamics of *Nb*SOBIR1 microdomains on the plasma membrane, including its lateral movement and downregulation *via* endocytosis, is regulated through the remodeling of cortical ER underneath the plasma membrane (Figures 4, 5). We suspect that the *Nb*RLP1 and *Nb*SOBIR1 interaction may contribute to binding the two compartments together to enable the coupled motility. However, when we performed biochemical studies to learn about the contribution of *Nb*RLP1 domains to the *Nb*RLP1-*Nb*SOBIR1 complex formation, the data suggested that the interaction between *Nb*RLP1 and *Nb*SOBIR1 might be bridged by other yet undiscovered factors (Supplementary Figure 2). Further evidence indicated that *Nb*RLP1, devoid of its cytoplasmic tail, showed slightly compromised activity to downregulate the number of *Nb*SOBIR1 microdomains on the plasma membrane (Supplementary Figure 2C), despite its binding with *Nb*SOBIR1. These observations have prompted us to hypothesize that the interaction between *Nb*RLP1 and *Nb*SOBIR1 is achieved transiently and/or involves remodeling of a complicated protein complex in order to regulate *Nb*SOBIR1 endocytosis. Along this line, understanding the exact composition of the *Nb*RLP1 and *Nb*SOBIR1-associated complex would be necessary to know how the ER contributes to *Nb*SOBIR1-mediated PTI response and *Nb*SOBIR1 endocytosis.

The *Nb*SOBIR1 microdomains in our studies showed an interesting pattern of either abutting or surrounded by the ER (Figure 5). In contrast to the flat ER sheets, the ER tubules are highly curved structures, shaped by the evolutionarily conserved reticulon family proteins that adopt a wedge-like topology in the ER. The reticulons localized to the border of the ER sheets, the ER tubules, and the three-way junction, all of which define the areas of the ER membrane that are highly curved (Nziengui et al., 2007). Intriguingly, these areas appear to be preferred by the *Nb*SOBIR1 microdomains on the plasma membrane to associate with the ER. Recently, EPCSs marked by SYT1-mCherry are identified at static ER tubules in plant (Ishikawa et al., 2018). In *Arabidopsis thaliana*, *At*SYT1 plays an essential role in maintaining cell integrity and virus movement (Uchiyama et al., 2014; Perez-Sancho et al., 2015). Notably, another plant EPCS tether *At*VAP27-1 has recently been shown to bind clathrin and phosphoinositides in *Arabidopsis* and *vap27-1/-3* mutant showed endocytosis defects, thus raising the possibility that endocytosis may occur at EPCS sites in plants (Stefano et al., 2018). In non-plant systems, EPCSs have been reported to get involved in diverse functions including lipid homeostasis, calcium influx, signaling, and endocytosis (Wakana et al., 2015; van der Burgh et al., 2019). Although the relationship between EPCSs and plant PRRs has not been established, the changes of EPCS in space and time in response to pathogen infection are definitely of great interest to be explored as a missing link in plant immunity.

Given that *Nb*SOBIR1 moves from the plasma membrane to endocytic vesicles in response to the ParA1 elicitin (Peng et al., 2015), it seems most likely that *Nb*RLP1 might regulate *Nb*SOBIR1 endocytosis. Indeed, overexpressing of *Nb*RLP1 in the absence of ParA1 treatment significantly reduced the number of *Nb*SOBIR1 microdomains on the plasma membrane (Figure 6D). In the presence of ParA1, *Nb*RLP1 overexpression though showed no effect on the total number of *Nb*SOBIR1 microdomains on the plasma membrane (Figure 6E, left) significantly accelerated the mobility of the ParA1-induced microdomains (Figure 6F) and ParA1-triggered *Nb*SOBIR1 endocytosis (Figure 6E, right). The SOBIR1 is involved in PTI elicited by various proteinaceous MAMPs. Whether *Nb*RLP1 similarly regulates *Nb*SOBIR1 endocytosis that is triggered by other elicitors await further investigation. It would also be interesting to know whether endocytosis of the corresponding RLP-PRRs is regulated by *Nb*RLP1 and exactly how endocytosis regulation is accomplished in this scenario.

In this study, we detected on the immune blot, two forms of *Nb*SOBIR1 which differ in molecular weight of around ∼8.5 kD (Figure 3B), implying post-translational protein modification on *Nb*SOBIR1. Interestingly, overexpression of *Nb*RLP1 resulted in the reduction of the higher molecular weight form of *Nb*SOBIR1 that is also the form that binds *Nb*RLP1 (Figure 3C). Our hunch is that the *Nb*SOBIR1 of greater molecular weight may reside in the microdomains to mediate *Nb*RLP1 interaction. Since *Nb*RLP1 overexpression reduced the *Nb*SOBIR1 microdomain on the plasma membrane (Figure 6D), through facilitating its endocytosis, it seems plausible to predict that the reduction in protein abundance is due to its endocytic turnover. It has been reported that SOBIR1 when overexpressed constitutively activates immune responses and is highly phosphorylated in *A. thaliana*, likely through the kinase activity of SOBIR1 itself and BAK1 (van der Burgh et al., 2019). However, using an antibody specifically against phosphoserine failed to recognize the *Nb*SOBIR1 pulled down by *Nb*RLP1-TAP (our unpublished data). In addition, the larger *Nb*SOBIR1 that binds *Nb*RLP1 also showed resistance to phosphatase treatment (our unpublished data). Thus, this unique form of *Nb*SOBIR1 does not seem to represent a phosphorylated and activated form of this protein kinase at least in *N. benthamiana*. It would be of great interest to further identify the PTM and know whether it plays an important regulatory role for MAMP perception and PTI response in plants.

As shown by phylogenetic analysis, *Nb*RLP1 clustered with two genes from *N. sylvestris* and *N. attenuata*, respectively. This clade is distinct from that encompassing EILP from *N. tabacum* (Takemoto et al., 2000) but closer to that containing two Cf-5 homologs (Hcr2-0A and Hcr2-0B) from *S. lycopersicum* (Dixon et al., 1998). Therefore, *Nb*RLP1 appears not as an ortholog of EILP. Regarding the plant defense response, we document that *Nb*RLP1 overexpression enhanced ParA1-induced necrosis (Figure 7). Although its silencing did not show a profound effect on ParA1-induced necrosis, SOBIR1 endocytosis, and plant resistance against *P. parasitica* (Supplementary Figure 3), a previous report showed overexpressing a fragment of *Nb*RLP1 (termed *Nb*EILP previously) in plants enhanced the accumulation of *Bamboo mosaic virus*, whereas gene silencing reduced its accumulation (Chen et al., 2017). Collectively, it seems likely that this ER RLP actively participates in a variety of host–pathogen interactions. It would be necessary to further investigate the exact molecular function of *Nb*RLP1 in the ER *in planta*.

Overall, our studies on *Nb*RLP1 and *Nb*SOBIR1 have provided many new insights into the tale of *Nb*SOBIR1-mediated PTI. The discovery of the *Nb*SOBIR1 microdomain at the plasma membrane and the control of its dynamics through the contact with the ER not only advance our current knowledge on this important adaptor protein but also reinforce the concept of microdomain formation as an important platform during MAMP perception. Though more needs to be learned from how SOBIR1 works in concert with various RLPs to achieve plant immunity, it seems equally important to think beyond the plasma membrane toward a potential role of ER either in transmitting plant immunity signals or even in the organization of immune receptor complex upon MAMP perception.

## Materials and Methods

### Plant Growth and Pathogen Culture Conditions

*Nicotiana benthamiana* was grown in a mixture of peat moss, perlite, and vermiculite (4:1:1) at 28°C under 12-h light/dark. *Phytophthora parasitica* (isolate 94069) was cultured on 10% V8 juice agar [10% V8 juice (Campbell, NJ, United States), 0.02% CaCO_3_, and 1.5% select agar (Thermo Fisher Scientific, Waltham, MA, United States)] at 25°C.

### Cloning and Sequence Analysis of *Nb*RLP1

Total RNAs were isolated from *N. benthamiana* leaves using the Plant Total RNA extraction kit (Viogene, New Taipei City, Taiwan) followed by using Turbo-DNA-free kit (Thermo Fisher Scientific, Waltham, MA, United States) to remove the residual DNA. *N. benthamiana* complementary DNA (cDNA) was prepared by using SuperScript III reverse transcriptase (Thermo Fisher Scientific, Waltham, MA, United States). The *NbRLP1* was amplified by using NbRLP1_F1-3 and NbRLP1_R1-2 primers (Supplementary Table 1), followed by cloning into the pENTR^TM^/D-TOPO^TM^ vector (Thermo Fisher Scientific, Waltham, MA, United States) for sequencing and subsequent subcloning. The signal peptide of *Nb*RLP1 was predicted by using Signal P-5.0^2^. Positions of leucine-rich repeat and transmembrane domain of *Nb*RLP1 were predicted by InterPro^3^ and TMHMM Server v.2.0^4^, respectively. Multiple sequence alignment involved the use of Clustal X. Phylogenetic tree was generated by the maximum likelihood algorithm implemented in MEGA (v.10.0.5) with the default parameters. Nodal support of the tree was estimated by bootstrapping with 1,000 pseudoreplicate data sets.

### Gene Expression Levels in the Infected Plants Quantified by qRT-PCR

At 0, 3, 6, 12, 24, 36, and 48 h after inoculation of *P. parasitica* zoospores, total RNAs from the 7th and 8th leaves of *N. benthamiana* were isolated and cDNA was synthesized as described in the previous section. Quantitative PCR (qPCR) was performed using the Power SYBR Green PCR Master Mix (Thermo Fisher Scientific, Waltham, MA, United States) with the primers listed in Supplementary Table 1 by using the StepOnePlus real-time PCR system (Applied Biosystems, Foster City, CA, United States). Raw data were normalized to the level of *NbEF1*α (as an internal control) and displayed as a fold-change relative to the transcript level of mock-treated plants of the same time points.

### Virus-Induced Gene Silencing

Virus-induced gene silencing (VIGS) experiments were performed as described by Peng et al. (2015). Briefly, a fragment of *Nb*RLP1 amplified by PCR with primers listed in Supplementary Table 1 was cloned into pTRV2 (pYL279) by Gateway cloning (Thermo Fisher Scientific, Waltham, MA, United States) to generate TRV2:*Nb*RLP1, which was transformed into *Agrobacterium tumefaciens* GV3101 strain. After growth in Luria-Bertani (LB) broth amended with rifampicin and kanamycin at 28°C for 18 h, the bacteria were diluted with induction medium (10 mM MES, pH 5.6, 10 mM MgCl_2_, and 200 μM acetosyringone) to OD_600_ of 0.6, which was then mixed with an equal volume of GV3101 bacteria harboring TRV1 (pYL192). The mixture was infiltrated onto leaves of 18-day-old *N. benthamiana* seedlings by the use of 1-mL syringes. Downregulation of gene expression was verified by qRT-PCR at 21 days post-agroinfection.

### ParA1 Purification and Assays of ParA1-Induced Necrosis

Expression of His-tagged ParA1 in *Escherichia coli* and the subsequent protein purification were performed as described by Peng et al. (2015) and Hofzumahaus and Schallmey (2013) with some modifications. *Escherichia coli* strain C43 (DE) harboring pET-20b(+):ParA1 was grown in terrific broth (TB) [1.2% tryptone, 2.4% yeast extract, 0.5% glycerol, and 1 M TB salts (0.17 M KH_2_PO_4_ and 0.72 M K_2_HPO_4_)] at 37°C. When OD_600_ reached 1.0, 0.4 mM isopropyl β- d-1-thiogalactopyranoside (IPTG) was added and the bacteria culture was grown at 30°C with constant shaking at 150 rpm. After 24 h, bacteria were harvested and disrupted in a solution [50 mM potassium phosphate buffer (pH 6.5), 500 mM NaCl, and 10 mM imidazole] by the use of a high-pressure homogenizer (Avestin EF-C3, Ottawa, ON, Canada) and His-tagged ParA1 recombinant protein was purified with the use of Ni-NTA agarose according to the protocol of the manufacturer (Qiagen, Hilden, Düsseldorf, Germany). For the ParA1-induced necrosis assay, 0.3 μM recombinant protein in 10 mM MES (pH 5.6) was infiltrated onto leaves of 4.5-week-old *N. benthamiana*. After 24 h, the respective areas of infiltration and necrotic lesion for each leaf were measured by using the ImageJ software, and necrosis index was calculated accordingly by dividing the necrosis area with the infiltrated area.

### Plasmid Construction

All plasmid constructs used in this study were made by using the Gateway cloning system (Thermo Fisher Scientific, Waltham, MA, United States). To generate the *GFP-NbRLP1* construct, the nucleotide sequence of *NbRLP1*ORF in the pENTR plasmid was first changed from T^79^C^80^C^81^A^84^ to A^79^G^80^T^81^T^84^, which introduced a *Sca*I site, whereas maintaining the same amino acid sequence. Then, a GFP-encoding DNA sequence was inserted into the *Sca*I site, followed by amplification and subcloning of the *GFP-NbRLP1* fragment into the pK7WG2 vector (Karimi et al., 2002) by Gateway cloning to get *pK7WG2:GFP-NbRLP1*. To generate the *Nb*RLP1-ΔN construct, a DNA fragment encompassing nucleotides 2,737–2,910 of *NbRLP1* ORF was amplified by PCR, which was then used to replace *NbRLP1* devoid of the signal peptide in *pENTR:NbRLP1* and then to get *pK7FWG2:NbRLP1-*Δ*N* by Gateway cloning. To generate the *NbRLP1-GFP* and *NbRLP1-*Δ*C* constructs, DNA fragments corresponding to the full-length *NbRLP1* ORF or *NbRLP1* lacking its cytoplasmic tail (with nucleotides 1–2805) were amplified by PCR and cloned into *pK7FWG2* to get *pK7FWG2:NbRLP1* and *pK7FWG2:NbRLP1-*Δ*C*, respectively. To prepare the constructs for the BiFC experiments, fragments of Venus N-terminal half (Vn) and Venus C-terminal half (Vc) were amplified (Sung and Huh, 2007) using primers listed in Supplementary Table 1 and cloned into *pENTR-NbRLP1* and *pENTR-NbSOBIR1* to get *pK7WG2:NbRLP1-Vn* and *pK7WG2:NbSOBIR1-Vc*, respectively. To get *pK7WG2:Vn-NbRLP1*, Vn fragment was amplified to replace GFP in *pENTR:GFP-NbRLP1* followed by Gateway cloning. To get TAP-tagged expression constructs, TAP sequence (Puig et al., 2001) synthesized by Genomics (Xizhi, New Taipei City, Taiwan) was first cloned to *pENTR:NbRLP1*, *pENTR:NbRLP1-*Δ*N*, and *pENTR:NbRLP1-*Δ*C*, respectively, followed by Gateway cloning. To prepare the *AtSYT1-mCherry* construct, the *AtSYT1* amplified from the cDNA of *A. thaliana* was cloned into *p35S-C-mCherry* (Wu et al., 2011), followed by PCR amplification of the *AtSYT1-mCherry* fragment and subcloned into *pK7WG2*. The sequences for all primers are listed in Supplementary Table 1.

### Confocal Imaging

For transient gene expression on *N. benthamiana*, the plasmid constructs described in the section above were transformed into *Agrobacterium tumefaciens* C58C1 for agroinfiltration as described by Peng et al. (2015). In brief, *A. tumefaciens* strains carrying constructs were cultured in LB broth amended with corresponding antibiotic at 28°C. After 18 h, the bacteria cells were harvested by centrifugation, resuspended with MMA (10 mM MES, pH 5.6, 10 mM MgCl_2_, 200 μM acetosyringone) to OD_600_ of 0.2 for *Nb*RLP1-GFP, 0.05 for *Nb*SOBIR1-mCherry, and 0.2 for *At*SYT1-mCherry, respectively. Bacteria were infiltrated onto the newly expanding leaves of 4-week-old *N. benthamiana* seedlings by the use of 1-mL needleless syringes. To suppress the silencing response, all treatments were coinfiltrated with *A. tumefaciens* C58C1 harboring P19-expressing construct (OD_600_of 0.1). For the BiFC experiments, *A. tumefaciens* C58C1 carrying *pK7WG2:NbSOBIR1-Vc* or *pK7WG2:Vc* was adjusted to OD_600_ of 0.05 and those carrying *pK7WG2:Vn-NbRLP1*, *pK7WG2:NbRLP1-Vn*, or *pK7WG2:Vn* were adjusted to OD_600_ of 0.2. Fluorescence signals were visualized using the Zeiss LSM 510 Meta confocal microscope, Zeiss LSM 880 confocal microscope, or Leica Stellaris 8 confocal microscopy. The Zeiss LSM 880 confocal microscope with the Airyscan super resolution mode was used for imaging microdomains on the plasma membrane. GFP: excitation of 488 nm and emission from 500 to 550 nm; mCherry: excitation of 543 nm and emission from 565 to 615 nm; YFP: excitation of 514 nm and emission from 510 to 560 nm.

### Quantification of *Nb*SOBIR1 Microdomains and Endocytic Structures

To quantify *Nb*SOBIR1 microdomains, we took still images specifically focused on the plasma membrane section using the Zeiss LSM880 with Airyscan mode. We used the ImageJ freehand selection tool followed by the ROI manager tool to define and calculate the area of the focused plasma membrane region. We manually counted the number of microdomains (usually ∼0.4–0.8 μm) using the multi-points tool of ImageJ. The number of *Nb*SOBIR1 puncta on plasma membrane was calculated using the number of microdomains divided by the area of the plasma membrane. To analyze the dynamics of *Nb*SOBIR1 microdomains, we performed particle tracking analysis using the ImageJ plugin Trackmate. We selected LoG detector tool to analyze particle diameter and intensity with estimated blob diameter of 0.8 μm and a threshold of 6.0. For analyses, we selected HyperStack displayer as a viewer and the simple lack tracker tool with the setting of linking max distance of 8 μm, gap-closing max distance of 8 μm, and gap-closing frame gap 2. To quantify endocytic structures, five z-stack images beneath the plasma membrane, each with 2.05 μm overlapped, were maximally projected to a final stack size of 8.20 μm. We used ImageJ to define and calculate the selected ROI area, followed by manually counting the number of endocytic structures. The number of *Nb*SOBIR1 endocytic vesicles was calculated using the number of endocytic structures divided by the volume of the stack.

### TAP Purification

*Agrobacterium tumefaciens* C58C1 carrying *pK7WG2:NbRLP1-TAP*, *pK7WG2:NbRLP1-*Δ*N-TAP*, or *pK7WG2:NbRLP1-*Δ*C-TAP* (at a final OD_600_ of 0.2) was mixed with C58C1 harboring *pK7WG2:NbSOBIR1-mCherry* (at a final OD_600_ of 0.05) and infiltrated onto young expanding leaves of 4-week-old *N. benthamiana*. After 46 h, 1.5 g leaves were ground into fine powder in the presence of liquid nitrogen, followed by resuspension in 3 mL GTEN buffer (10% glycerol, 25 mM Tris–HCl, pH 7.5, 1 mM EDTA, 150 mM NaCl, 10 mM DTT, and 1.5% triton-X100) (Sacco et al., 2007) supplemented with protease inhibitors cocktail (Roche Molecular Systems, NJ, United States). After thawed, debris present in the mixture was removed by a quick spin of 1,000 rpm for 1 min, followed by another spin of 12,000 g for 10 min, both at 4°C. The resulting supernatant was collected and incubated with 100 μL IgG Sepharose^TM^ 6 FF at 4°C with gentle rotation for 3 h. After washing the beads four times with 10 ml GTEN buffer containing 1.25 mM PMSF, the bound proteins were eluted with 80 μL MURB (50 mM sodium phosphate, 25 mM MES, pH 7.0, 1% SDS, 3 M urea, and 5% β-mercaptoethanol) and boiled for 5 min. Samples were subjected to SDS-PAGE followed by the Western blot analysis with the use of PAP antibody (Jackson immune research, PA, United States) or our homemade anti-mCherry antibody (Wu et al., 2011). Signals on the blots were detected by using the UVP ChemiDoc-It imager (Upland, CA, United States).

### Inoculation of *P. parasitica*

For inoculation, zoospore suspension (2^∗^10^4^ spores/mL) prepared as described by Yan and Liou (2006), or water as controls, was sprayed evenly on 5.5-week-old *N. benthamiana* till run-off, and the inoculated plants were maintained at 28°C in the dark in a moisture chamber for the indicated periods of time. Disease severity was scored, as follows, on a scale from 0 to 4, according to symptoms developed on the plants: 0, healthy, no water-soaking lesions; 1, slight water-soaking, with less than 50% wilting leaves; 2, obvious water-soaking lesions, with more than 50% wilting leaves; 3, severe water-soaking and wilting; 4, complete wilting along with the appearance of mycelia. The disease severity index was calculated as [sum (number of plants × disease index)])/[(total number of plants) × (maximal disease index)] × 100.

### Statistical Analysis

All experiments were repeated at least three times. The two-tail student’s *t*-test was used for two-paired comparison, and the *p*-value was displayed. Multiple comparison analysis testing was carried out with ANOVA and LSD (*p* < 0.05).

## Data Availability Statement

The original contributions presented in the study are publicly available. This data can be found here: The 2.91- and 1.41-kb NbRLP1 sequences can be found in the GenBank under the following accession numbers: MW924093 and MW924094, respectively.

## Author Contributions

R-FL and C-WW planned, designed the research, and wrote the manuscript. Y-HL, W-CS, and T-YK executed the experiments and analyzed the data. All authors contributed to the article and approved the submitted version.

## Conflict of Interest

The authors declare that the research was conducted in the absence of any commercial or financial relationships that could be construed as a potential conflict of interest.

## Publisher’s Note

All claims expressed in this article are solely those of the authors and do not necessarily represent those of their affiliated organizations, or those of the publisher, the editors and the reviewers. Any product that may be evaluated in this article, or claim that may be made by its manufacturer, is not guaranteed or endorsed by the publisher.

## Footnotes

1
http://solgenomics.net/

2
http://www.cbs.dtu.dk/services/SignalP/

3
http://www.ebi.ac.uk/interpro/

4
http://www.cbs.dtu.dk/services/TMHMM/

## Supplementary Material

Supplementary Figure 1 Colocalization of *Nb*RLP1-GFP and *At*SYT1-mCherry. *Nicotiana benthamiana* leaves expressing *Nb*RLP1-GFP and *At*SYT1-mCherry were imaged at the periphery sections using Leica Stellaris 8 confocal microscope. Scale bar, 10 μm. The box area in the image of the periphery section is enlarged to show the colocalization of *Nb*RLP1-GFP and *At*SYT1-mCherry signals.

Supplementary Figure 2 C-terminus of *Nb*RLP1 is sufficient for binding with *Nb*SOBIR1. **(A)** *Nicotiana benthamiana* leaves expressing *Nb*SOBIR1-mCherry and *Nb*RLP1-TAP, *Nb*RLP1-ΔN-TAP, *Nb*RLP1-ΔC-TAP, or TAP as indicated were harvested and lyzed. The lysate (input) was subjected to pull-down by IgG Sepharose as described in the Materials and Methods section. The input and the bound (pulled-down) fractions were subjected to SDS-PAGE followed by the Western blot analysis with use of anti-mCherry or PAP antibodies. **(B)** A scheme depicting the current model of how *Nb*RLP1-Vn in the ER might interact with *Nb*SOBIR1-Vc on the plasma membrane. X, Y, and Z indicate putative proteins that might bridge the *Nb*SOBIR1–*Nb*RLP1 interaction. **(C)** Deletion of the C-terminus but not N-terminus of *Nb*RLP1 showed slightly compromised activity to downregulate the number of *Nb*SOBIR1 microdomains on the plasma membrane. *N. benthamiana* leaves expressing *Nb*SOBIR1-mCherry alone or coexpressing with *Nb*RLP1-GFP, *Nb*RLP1-ΔN-GFP or *Nb*RLP1-ΔC-GFP were subjected to microscopy by using the Zeiss LSM880 confocal microscope with use of Airyscan. Microdomain number was quantified as described in the Materials and Methods section and shown as scatter plots. Different letters indicate significant differences based on LSD (*p* < 0.01).

Supplementary Figure 3 Endocytosis occurs directly *via* the translocation of *Nb*SOBIR1 microdomains on the plasma membrane. *Nicotiana benthamiana* leaves expressing *Nb*SOBIR1-mCherry were infiltrated with 0.3 μM ParA1 in MES buffer. After 30 min, leaves were subjected to time-lapse microscopy by focusing on a few microdomains at the center of the cell using the Zeiss LSM880 confocal microscope with an Airyscan. The total image acquisition length was 16 min with a time interval of 5 s. Representative area was selected for display by time course.

Supplementary Figure 4 *Nb*RLP1 silencing did not show a profound effect on *Sl*SOBIR1 endocytosis or plant resistance against *Phytophthora parasitica*. **(A)***Sl*SOBIR1-GFP was expressed on *Nicotiana benthamiana* leaves pre-silenced for *Nb*RLP1 expression (TRV-*Nb*RLP1) or infected with TRV-GFP as the control. Around 44 h post-agroinfiltration, the leaves were treated with 0.3 μM ParA1 in MES buffer for 30 min, followed by microscopy with five Z stack for a total of 8.20 stack size using Zeiss LSM 510 confocal microscope. The maximal projection images are shown. The number of internal *Nb*SOBIR1-mCherry puncta was quantified as described in the Materials and Methods section and displayed using a scatter plot. Statistical analysis was carried out with the two-tailed student’s *t*-test and the *p*-value is shown. **(B)** *N. benthamiana* silenced for *Nb*RLP1 expression (TRV-NbRLP1) or treated as the control carrying TRV-GFP were inoculated with zoospores of *P. parasitica*. At indicated hours post-inoculation (hpi), the development of disease symptoms was examined and the disease severity index was calculated as detailed in the Materials and methods section. The experiment has been repeated three times with similar results, and only one set of the experimental data is shown.

Supplementary Table 1 A list of primers used in this study and the sequence of 1.41- and 2.91-Kb *Nb*RLP1.

Supplementary Movie 1 *Nicotiana benthamiana* leaves expressing *Nb*SOBIR1-mCherry were subjected to time-lapse microscopy, focusing only on the periphery of the leaf epidermal cell, using Zeiss LSM880 confocal microscope with Airyscan. The total image acquisition length was 16 min with a time interval of 5 s.

Supplementary Movie 2 *Nicotiana benthamiana* leaves expressing *Nb*SOBIR1-mCherry and *Nb*RLP1-GFP were subjected to time-lapse microscopy, focusing only on the periphery of the leaf epidermal cell, using Zeiss LSM880 confocal microscope with Airyscan. The total image acquisition length was 16 min with a time interval of 5 s.

Supplementary Movie 3 *Nicotiana benthamiana* leaves expressing *Nb*SOBIR1-mCherry were treated with 0.3 μM ParA1 in MES buffer. After 30 min, the leaves were subjected to microscopy, focusing only on the periphery of the leaf epidermal cell, by Zeiss LSM880 confocal microscope with use of Airyscan. The total image acquisition length was 16 min with a time interval of 5 s.

Supplementary Movie 4 *Nicotiana benthamiana* leaves expressing *Nb*SOBIR1-mCherry and *Nb*RLP1-GFP were treated with 0.3 μM ParA1 in MES buffer. After 30 min, the leaves were subjected to microscopy, focusing only on the periphery of the leaf epidermal cell, by using Zeiss LSM880 confocal microscope with Airyscan. The total image acquisition length was 16 min with a time interval of 5 s.
