# Supplementary figures and images for: NbSOBIR1 Partitions Into Plasma Membrane Microdomains and Binds ER-Localized NbRLP1
Source: Front Plant Sci. 2021 Sep 1;12:721548. doi: 10.3389/fpls.2021.721548 (PMC8442688; doi:10.3389/fpls.2021.721548)

**Figure S1**

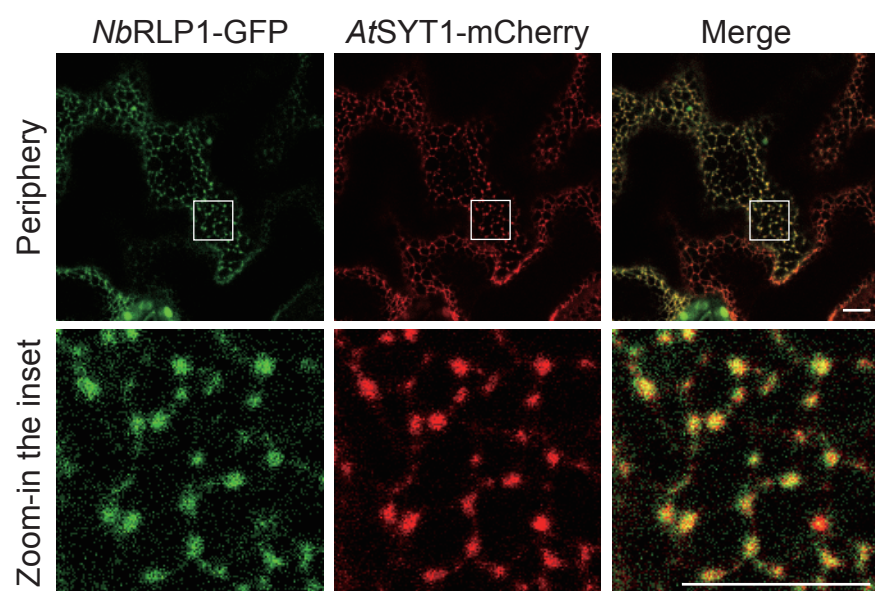

Supplement: Supplementary Figure 1 — Colocalization of NbRLP1-GFP and AtSYT1-mCherry. Nicotiana benthamiana leaves expressing NbRLP1-GFP and AtSYT1-mCherry were imaged at the periphery sections using Leica Stellaris 8 confocal microscope. Scale bar, 10 μm. The box area in the image of the periphery section is enlarged to show the colocalization of NbRLP1-GFP and AtSYT1-mCherry signals. [file Image_1.PDF]

**Figure S2**

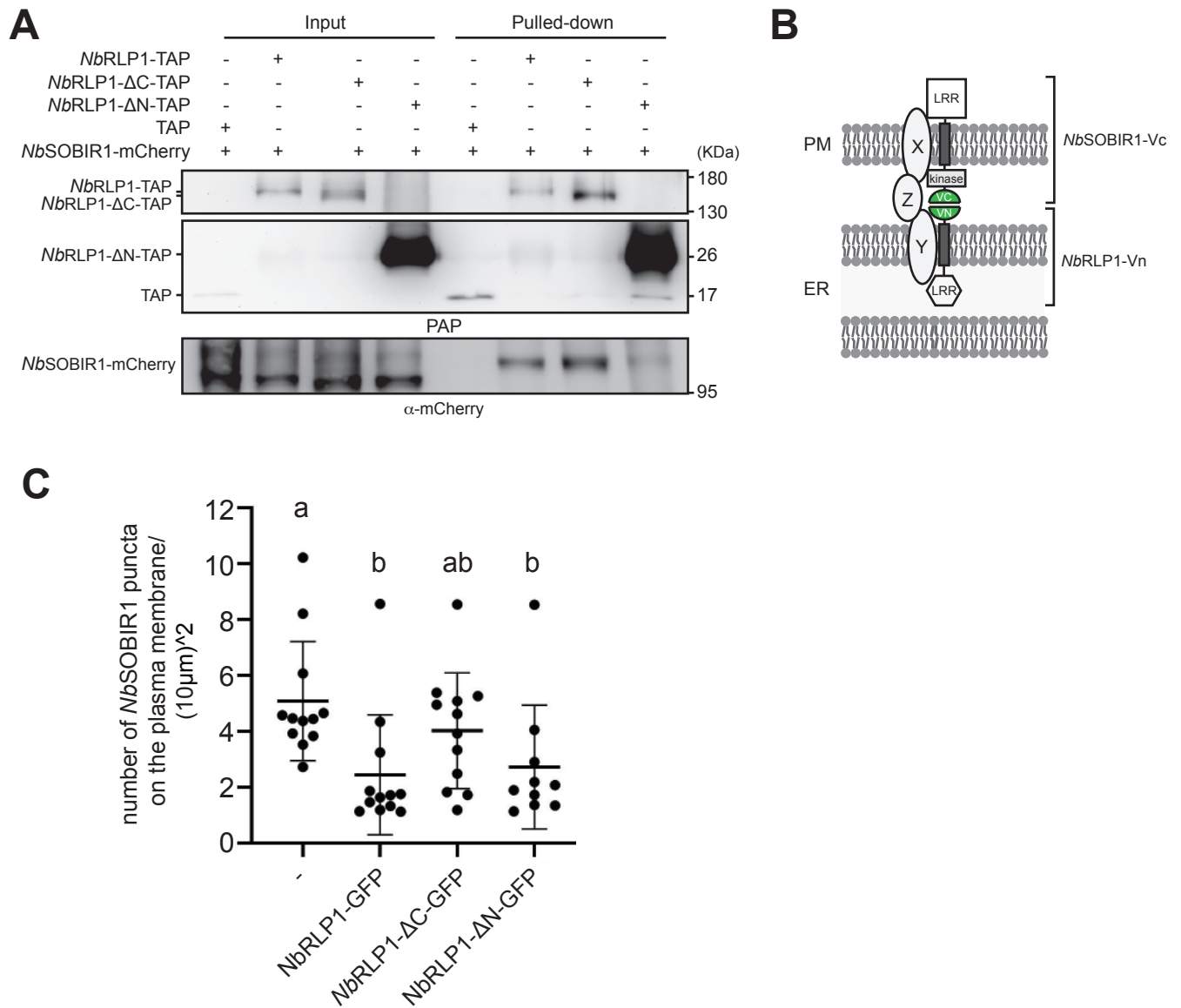

Supplement: Supplementary Figure 2 — C-terminus of NbRLP1 is sufficient for binding with NbSOBIR1. (A) Nicotiana benthamiana leaves expressing NbSOBIR1-mCherry and NbRLP1-TAP, NbRLP1-ΔN-TAP, NbRLP1-ΔC-TAP, or TAP as indicated were harvested and lyzed. The lysate (input) was subjected to pull-down by IgG Sepharose as described in the Materials and Methods section. The input and the bound (pulled-down) fractions were subjected to SDS-PAGE followed by the Western blot analysis with use of anti-mCherry or PAP antibodies. (B) A scheme depicting the current model of how NbRLP1-Vn in the ER might interact with NbSOBIR1-Vc on the plasma membrane. X, Y, and Z indicate putative proteins that might bridge the NbSOBIR1–NbRLP1 interaction. (C) Deletion of the C-terminus but not N-terminus of NbRLP1 showed slightly compromised activity to downregulate the number of NbSOBIR1 microdomains on the plasma membrane. N. benthamiana leaves expressing NbSOBIR1-mCherry alone or coexpressing with NbRLP1-GFP, NbRLP1-ΔN-GFP or NbRLP1-ΔC-GFP were subjected to microscopy by using the Zeiss LSM880 confocal microscope with use of Airyscan. Microdomain number was quantified as described in the Materials and Methods section and shown as scatter plots. Different letters indicate significant differences based on LSD (p < 0.01). [file Image_2.PDF]

Figure S4

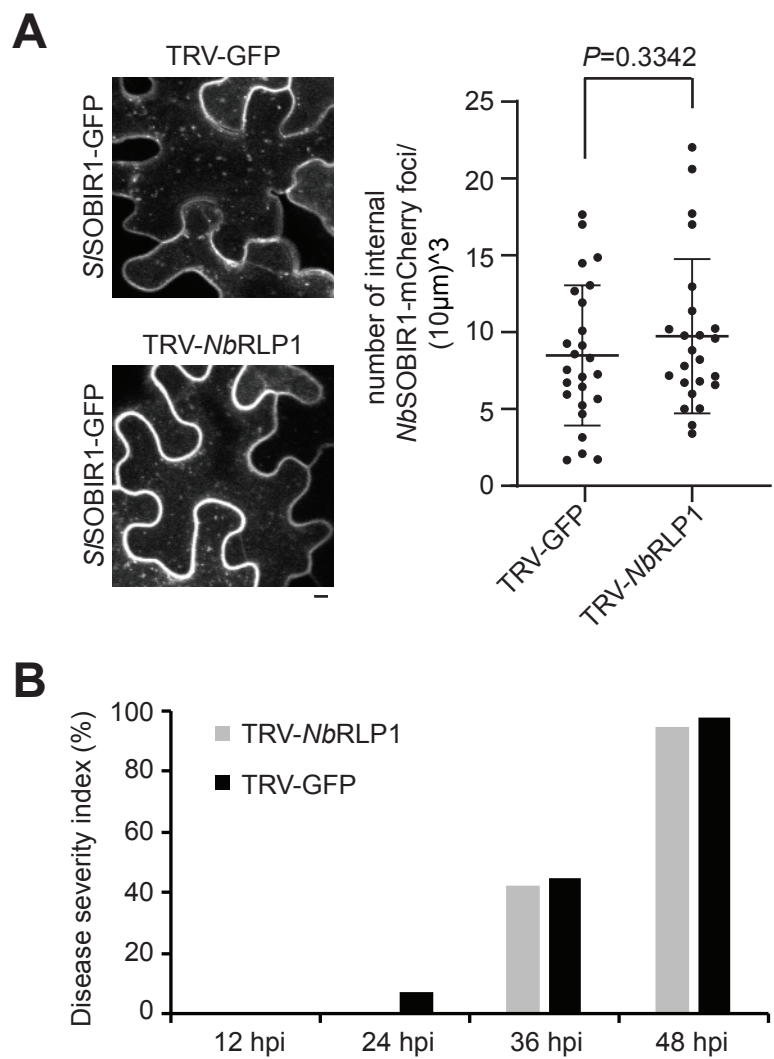

Supplement: Supplementary Figure 4 — NbRLP1 silencing did not show a profound effect on SlSOBIR1 endocytosis or plant resistance against Phytophthora parasitica. (A)SlSOBIR1-GFP was expressed on Nicotiana benthamiana leaves pre-silenced for NbRLP1 expression (TRV-NbRLP1) or infected with TRV-GFP as the control. Around 44 h post-agroinfiltration, the leaves were treated with 0.3 μM ParA1 in MES buffer for 30 min, followed by microscopy with five Z stack for a total of 8.20 stack size using Zeiss LSM 510 confocal microscope. The maximal projection images are shown. The number of internal NbSOBIR1-mCherry puncta was quantified as described in the Materials and Methods section and displayed using a scatter plot. Statistical analysis was carried out with the two-tailed student’s t-test and the p-value is shown. (B) N. benthamiana silenced for NbRLP1 expression (TRV-NbRLP1) or treated as the control carrying TRV-GFP were inoculated with zoospores of P. parasitica. At indicated hours post-inoculation (hpi), the development of disease symptoms was examined and the disease severity index was calculated as detailed in the Materials and methods section. The experiment has been repeated three times with similar results, and only one set of the experimental data is shown. [file Image_4.PDF]
