# Supplementary material for: NbSOBIR1 Partitions Into Plasma Membrane Microdomains and Binds ER-Localized NbRLP1
Source: Front Plant Sci. 2021 Sep 1;12:721548. doi: 10.3389/fpls.2021.721548 (PMC8442688; doi:10.3389/fpls.2021.721548)

Figure S3

(Example 1)

▲ *Nb*SOBIR1-mCherry microdomain undergoing endocytosis (blue)

► *Nb*SOBIR1-mCherry microdomain undergoing endocytosis (yellow)

► The development of *Nb*SOBIR1-mCherry microdomain (magenta)

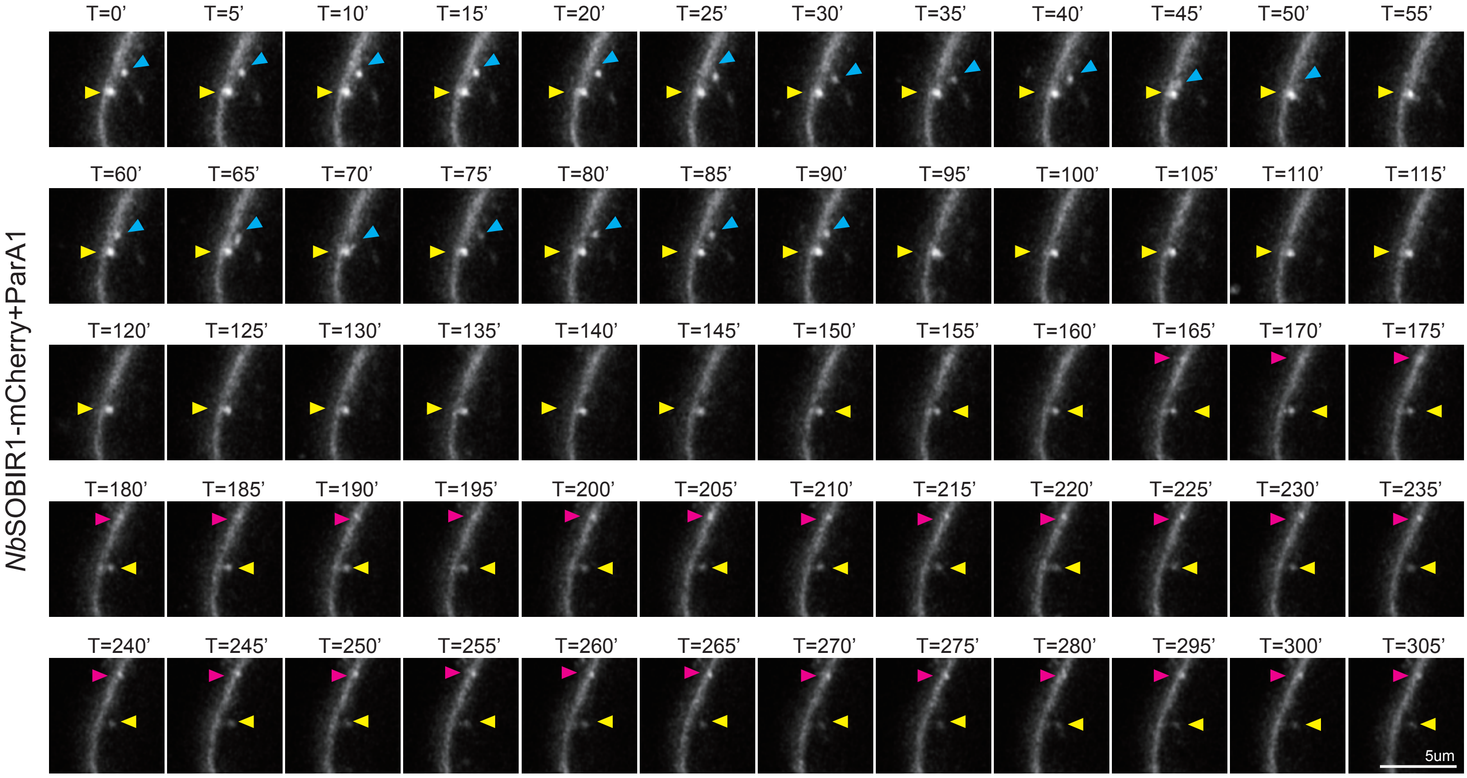

(Example 2)

▲ *Nb*SOBIR1-mCherry microdomain undergoing endocytosis (blue)

► *Nb*SOBIR1-mCherry microdomain undergoing endocytosis (yellow)

► The development of *Nb*SOBIR1-mCherry microdomain (magenta)

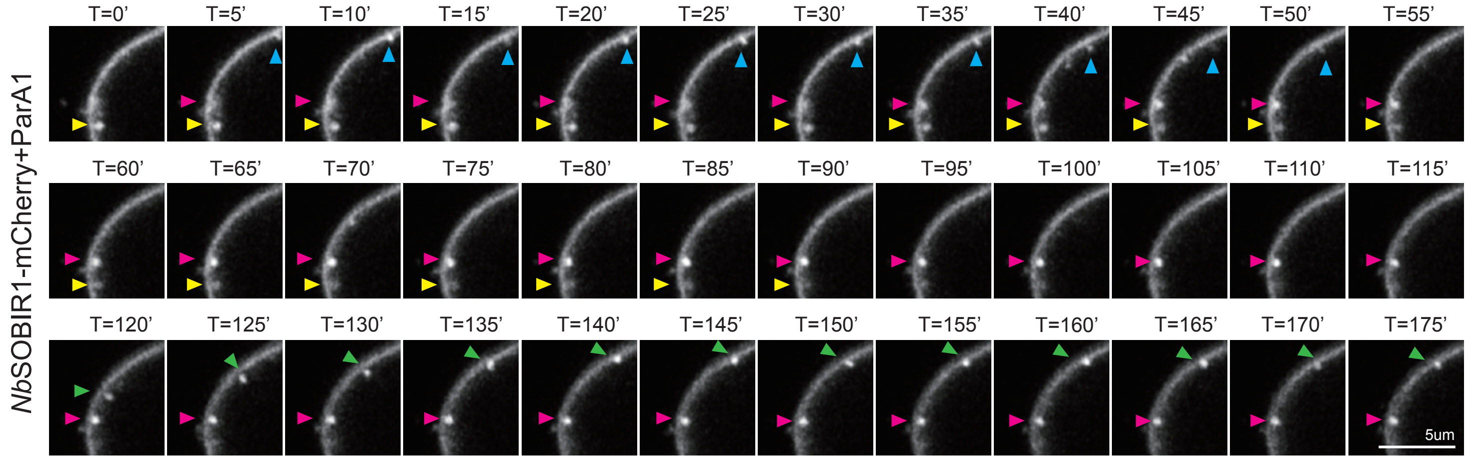

Supplement: Supplementary Figure 3 — Endocytosis occurs directly via the translocation of NbSOBIR1 microdomains on the plasma membrane. Nicotiana benthamiana leaves expressing NbSOBIR1-mCherry were infiltrated with 0.3 μM ParA1 in MES buffer. After 30 min, leaves were subjected to time-lapse microscopy by focusing on a few microdomains at the center of the cell using the Zeiss LSM880 confocal microscope with an Airyscan. The total image acquisition length was 16 min with a time interval of 5 s. Representative area was selected for display by time course. [file Image_3.PDF]
